# FGFR4 overexpression and hotspot mutations in metastatic ER+ breast cancer are enriched in the lobular subtype

**DOI:** 10.1038/s41523-019-0114-x

**Published:** 2019-06-27

**Authors:** Kevin M. Levine, Nolan Priedigkeit, Ahmed Basudan, Nilgun Tasdemir, Matthew J. Sikora, Ethan S. Sokol, Ryan J. Hartmaier, Kai Ding, Nedah Z. Ahmad, Rebecca J. Watters, Kurt R. Weiss, Jens-Uwe Blohmer, Carsten Denkert, Anna Machleidt, Maria M. Karsten, Michelle M. Boisen, Esther Elishaev, Peter C. Lucas, Adrian V. Lee, Steffi Oesterreich

**Affiliations:** 1 Women’s Cancer Research Center, UPMC Hillman Cancer Center, Pittsburgh, PA USA; 2 Magee-Women’s Research Institute, Magee-Women’s Research Hospital of University of Pittsburgh Medical Center, Pittsburgh, PA USA; 3 Medical Scientist Training Program, University of Pittsburgh School of Medicine, Pittsburgh, PA USA; 4 Department of Pathology, University of Pittsburgh, Pittsburgh, PA USA; 5 Department of Pharmacology and Chemical Biology, University of Pittsburgh, Pittsburgh, PA USA; 6 Department of Human Genetics, University of Pittsburgh, Pittsburgh, PA USA; 7 Department of Pathology, University of Colorado Anschutz Medical Campus, Aurora, CO USA; 8 Foundation Medicine, Cambridge, MA USA; 9 Integrative Systems Biology Program, University of Pittsburgh, Pittsburgh, PA USA; 10 Department of Orthopedic Surgery, University of Pittsburgh, Pittsburgh, PA USA; 11 Charité-Universitätsmedizin Berlin, Berlin, Germany; 12 Department of Obstetrics, Gynecology, & Reproductive Sciences, University of Pittsburgh, Pittsburgh, PA USA; 13 Present Address: Department of Clinical Laboratory Sciences, King Saud University, Riyadh, Saudi Arabia

**Keywords:** Breast cancer, Metastasis

## Abstract

Invasive lobular carcinoma (ILC) is an understudied subtype of breast cancer that requires novel therapies in the advanced setting. To study acquired resistance to endocrine therapy in ILC, we have recently performed RNA-Sequencing on long-term estrogen deprived cell lines and identified FGFR4 overexpression as a top druggable target. Here, we show that FGFR4 expression also increases dramatically in endocrine-treated distant metastases, with an average fold change of 4.8 relative to the paired primary breast tumor for ILC, and 2.4-fold for invasive ductal carcinoma (IDC). In addition, we now report that FGFR4 hotspot mutations are enriched in metastatic breast cancer, with an additional enrichment for ILC, suggesting a multimodal selection of FGFR4 activation. These data collectively support the notion that FGFR4 is an important mediator of endocrine resistance in ILC, warranting future mechanistic studies on downstream signaling of overexpressed wild-type and mutant FGFR4.

## Introduction

Invasive lobular carcinoma (ILC) is a common histological subtype, accounting for 10–15% of all breast cancer diagnoses. Since most of these tumors are estrogen receptor positive (ER+), patients with ILC are often treated with endocrine therapy. Although these treatments are highly efficacious for most patients initially, long-term recurrences remain a major clinical problem for ILC.^1,2^ We have recently performed RNA-Sequencing on paired, metachronous primary, and metastatic tumors to the brain and bone.^3,4^ Here, we perform a subset analysis on the previously published clinical data, focusing only on ER+ patients treated with endocrine therapy prior to their recurrence, as well as report additional FGFR4 expression data from paired gastrointestinal (GI) and ovarian metastases.^5^

## Results

### FGFR4 overexpression in endocrine-treated cell lines

**Fig. 1:**
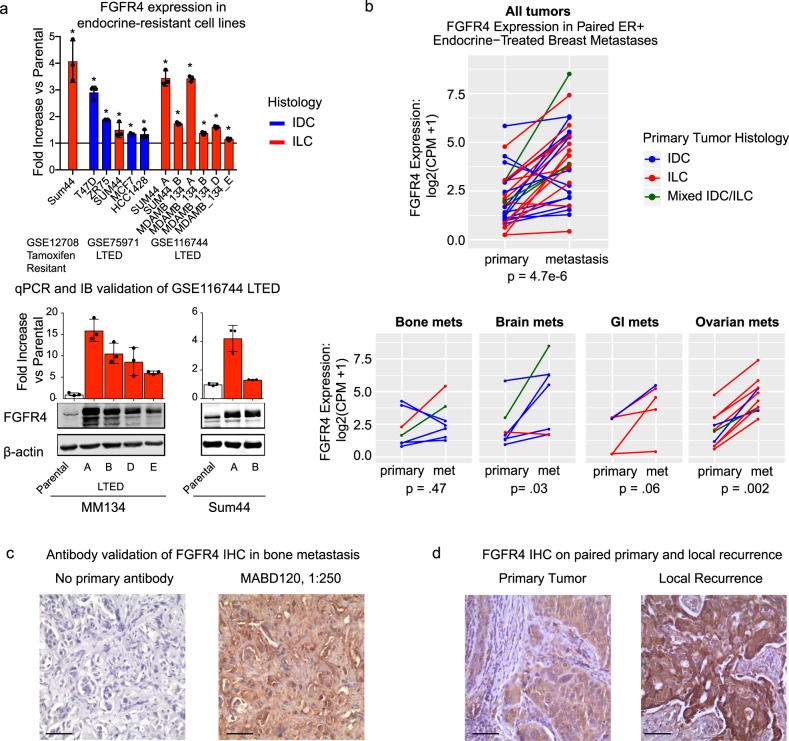
FGFR4 expression is elevated in cell lines and patient samples treated with endocrine therapy. **a** Top, FGFR4 RNA expression fold-change in long-term endocrine-resistant cell line models of ER+ breast cancer, relative to parental cell lines with short-term estrogen deprivation. From left to right, tamoxifen-resistant cells from GSE12708, long-term estrogen deprived (LTED) cells from GSE75971, LTED cells from GSE116744. *p < 0.05 for differential expression versus parental, corrected for multiple comparisons with Benjamini–Hochberg. Bottom, qRT-PCR and immunoblot comparison of FGFR4 expression in MM134 and SUM44 LTED cells, relative to parental cells grown in FBS. Error bars represent ± SD for three biological replicates. Red bars represent ILC and blue bars represent IDC. **b** Top, FGFR4 expression gain in 29 ER+ paired tumors. Bottom, FGFR4 expression gain in the tumors separated by site of metastasis (met). Red lines represent primary tumor histology of ILC, blue lines represent IDC, and green lines represent mixed IDC/ILC tumors. Two-sided paired Wilcoxon rank tests were used to calculate *p* values for FGFR4 gain. **c** IHC staining of an orphan bone metastasis (left, no primary antibody. right, FGFR4 (MABD120, 1:250 dilution)). **d** IHC staining of FGFR4 (MABD120, 1:250 dilution) in a paired primary breast tumor and endocrine-treated local recurrence. Scale bars represent 100 µm. See Supplementary Material for additional antibody validation

To model acquired resistance to endocrine therapy in the laboratory, we and others have recently performed RNA-Sequencing on long-term estrogen deprived (LTED) cell lines (GSE116744^6^ and GSE75971^7^) or microarray analysis on tamoxifen resistant cell lines (GSE12708^8^). FGFR4 is overexpressed in 8/8 ILC cell line models and 4/4 invasive ductal carcinoma (IDC) cell line models at the RNA level relative to parental cells subjected to short-term estrogen deprivation (Fig. 1a, top panel). Importantly, the FGFR4 overexpression in our ILC LTED cells was also observed relative to parental cells in full serum, at the RNA and protein level (Fig. 1a, bottom panel).

### FGFR4 overexpression in endocrine-treated metastases

To investigate the clinical relevance of this finding, FGFR4 RNA expression was next examined in our previous and ongoing studies of paired primary and metastatic tumors. In this subset analysis, we focus on the patients with ER+ primary tumors who received endocrine therapy prior to the recurrence of bone,^3^ brain,^4^ or GI/ovarian metastases.^5^ From a total of 26 patients, we collected treatment-naïve primary tumors and 29 endocrine-treated metastases, consisting of 7 bone, 7 brain, 5 GI, and 10 ovarian metastases. The average time to recurrence from primary to matched metastasis was 59 months. Our study cohorts were enriched for ILC, and this subset analysis consists of a histological distribution of 13 IDCs, 13 ILCs, and 3 cases of mixed IDC/ILC. Overall, 26/29 (90%) metastases have an increase in FGFR4 RNA relative to their matched primary tumor (*p* = 4.7e−6), including 19/29 (66%) with a fold change >2 (Fig. 1b, top panel). Of note, there were patients with large gains in FGFR4 across all four distant metastatic sites studied, with significant gains in brain and ovarian metastases (Fig. 1b, bottom panel). Because of the small sample size for each metastatic site, there is no significant difference for FGFR4 expression gain by tumor site or histological type, nor is there an interaction effect (*p* > 0.05 for all three tests by two-way ANOVA). However, there is a trend for increased FGFR4 gain in ILC, with a mean increase of 4.8-fold for the ILCs versus 2.4-fold for the IDCs. We have validated an antibody for immunohistochemistry (IHC) detection of FGFR4 protein expression and have preliminary data for staining primary and recurrent breast tissue (Supplementary Material, Fig. 1c, d).

### FGFR4 hotspot mutations are enriched in metastatic ER+ ILC

**Fig. 2:**
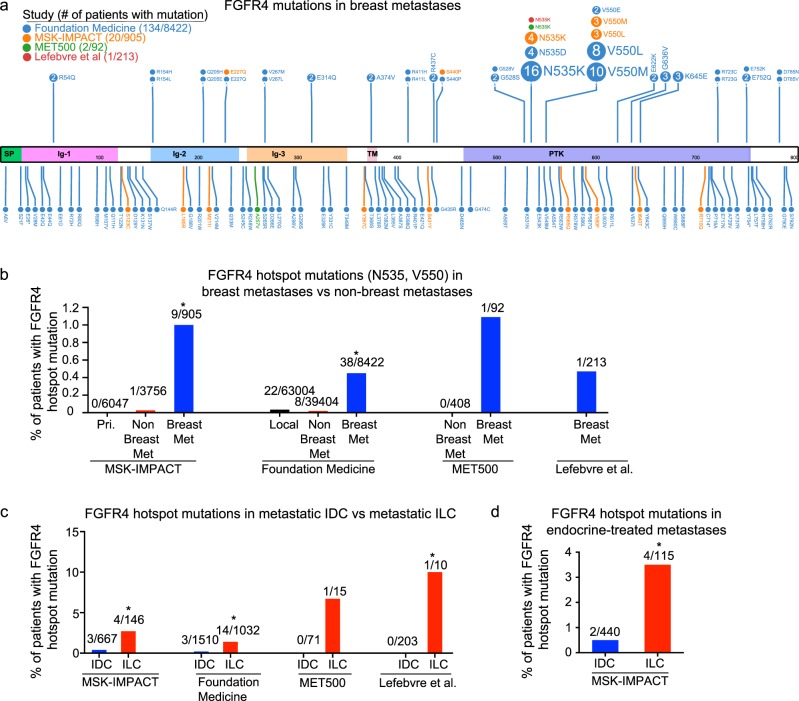
FGFR4 hotspot (N535 and V550) mutations are enriched in metastatic ILC. **a** Lollipop plot of FGFR4 mutations generated using ProteinPaint.^31^ Top: all mutations appearing at least twice. **b** FGFR4 hotspot (N535 and V550) mutations in MSK-IMPACT primary (pri.), nonbreast metastatic (met), and breast metastatic tumors, Foundation Medicine local, nonbreast metastatic, and breast metastatic tumors, MET500 nonbreast and breast metastatic, and Lefebvre et al. breast metastatic tumors. *FGFR4 hotspot mutations are enriched in breast metastatic tumors versus nonbreast metastatic tumors (MSK-IMPACT OR: 38.7, Fisher exact *p* = 5.8e-6, Foundation Medicine OR = 22.3, Fisher exact *p* < 2.2e−16). **c** FGFR4 hotspot mutations in metastatic ILC versus metastatic IDC. *FGFR4 hotspot mutations are enriched in ILC (MSK-IMPACT: OR = 6.2, *p* = 0.02, Foundation Medicine: OR = 6.9, *p* < 0.0007, Lefebvre et al.: OR = Inf., *p* = 0.05). **d** FGFR4 hotspot mutations in endocrine-treated metastatic ILC versus metastatic IDC. *FGFR4 hotspot mutations are enriched in ILC (MSK-IMPACT: OR = 7.9, *p* = 0.02)

Next, the rate of FGFR4 mutations in metastatic cancer was examined in all patients from three recent sequencing studies: MSK-IMPACT,^9,10^ MET500,^11^ and Lefebvre et al.,^12^ as well as from sequencing data from Foundation Medicine. Figure 2a shows the distribution of FGFR4 mutations in these studies, with the most frequently mutated sites being the FGFR4 hotspot mutations previously identified in rhabdomyosarcomas (N535 and V550).^13–15^ Although FGFR4 hotspot mutations are rarely detected in primary tumors (<0.05%), they are present in ~0.5–1% of breast metastases, significantly enriched relative to nonbreast metastases (~0.02%) (Fig. 2b). In addition, these hotspot mutations are enriched significantly in metastatic ILC relative to metastatic IDC (Fig. 2c). Treatment data is only available for the MSK-IMPACT data, which shows that 8/9 patients with FGFR4 hotspot mutations were previously treated with endocrine therapy (Supplementary Data 2). The total rate of FGFR4 hotspot mutations in patients with endocrine-treated metastases is 3.5% for ILC versus 0.5% for IDC (Fig. 2d). There are no mutations or copy-number alterations that significantly co-occur or are mutually exclusive with the FGFR4 hotspot mutations in patients with endocrine-treated metastases (*q* > 0.1).

## Discussion

In our analyses of cell line models of acquired endocrine resistance, as well as clinical samples from pre- and post-endocrine treatment, we find that FGFR4 overexpression is a remarkably common phenomenon. FGFR4 overexpression in endocrine-resistant cell lines is seen relative to parental cells subjected to short-term estrogen deprivation, suggesting that the FGFR4 gains are not an artifact of estrogen loss. FGFR4 overexpression is also seen relative to parental cells growing in full serum, suggesting that FGFR4 is not simply a marker of proliferation. Instead, FGFR4 overexpression may represent a long-term signaling adaption in tumor cells following endocrine therapy. Studies are currently ongoing to assess possible causes of FGFR4 overexpression, the functional impact of FGFR4 inhibition, and the signaling mechanisms downstream of FGFR4. Recently, FGFR4 has been shown to activate AKT^16^ and inhibit MST1/2^17^ signaling in breast cancer cells, but the role of FGFR4 in ILC remains uncertain. Given that recent studies show that loss of E-cadherin expression can directly drive increased growth-factor receptor signaling in ILC, future studies will examine the interaction between E-cadherin and FGFR4.^18,19^

In our analyses of clinical specimens, we observed that the large gains in FGFR4 spanned all four distant metastatic sites. This data, as well as the fact that the brain, GI, and ovarian metastases underwent macrodissection prior to RNA extraction, suggest that the gains in FGFR4 are a result of overexpression within tumor cells. Tumor cell expression of FGFR4 is confirmed in our preliminary IHC analysis, but more samples are needed to fully assess the correlation between RNA and protein levels in metastatic tissue and the contribution of FGFR4 RNA from stromal cells.

The MSK-IMPACT data include copy-number analysis, which finds a low rate of FGFR4 DNA amplification (2/596, 0.3%) in endocrine-treated metastases, suggesting that copy-number gains are unlikely to account for the high rate of overexpression seen in our paired samples. Additional studies that contain both DNA and RNA analysis will be needed to assess if mutated FGFR4 is overexpressed.

Because of the low rate of mutations, copy number amplifications, and fusions of FGFR4 identified in previous studies, FGFR4 has been understudied in clinical trials relative to the other FGFR family members.^20^ However, there are clinical trials with novel pan-FGFR inhibitors that have high potency for wild-type and/or mutated FGFR4^21,22^ (NCT03238196), as well as at least four ongoing clinical trials with FGFR4-specific small molecules (NCT02325739, NCT02834780, NCT03144661, and NCT02508467). These FGFR4-specific inhibitors exhibit their specificity by interacting with a cysteine residue near the hotspot mutations, suggesting that although they are appropriate for wild-type overexpression of FGFR4, modifications would likely be needed to treat patients with hotspot mutations.^23^ Recent studies show that FGFR1 amplification may play a role in endocrine resistance, and that combined FGFR1 and CDK4/6 inhibition can reverse this phenotype.^24,25^ Future studies of resistance to combined endocrine therapy and CDK4/6 inhibition would benefit from evaluating FGFR4 overexpression and mutations as potential resistance factors, particularly for patients with lobular carcinoma.

## Methods

### RNA-Sequencing

RNA extraction and sequencing for the GI and ovarian metastases was performed as previously described for our brain and bone metastases cohorts.^3,4^ Briefly, biospecimens were reviewed by a trained molecular pathologist to confirm pathology, quantify tumor cellularity, and to highlight regions of relatively high tumor cellularity for macrodissection. RNA was extracted from FFPE tissue using Qiagen’s All-Prep Kit, and library preparation performed using Illumina’s TruSeq RNA Access Library Preparation protocol. Transcript counts from all samples were quantified with Salmon^26^ v.0.8.2 and converted to gene-level counts with tximport.^27^ The gene-level counts from all studies were then normalized together using TMM with edgeR.^28^ Log2 transformed TMM-normalized counts per million: log2 (TMM-CPM+1) expression values were used for the analysis. Collection and analysis of specimens was approved under the University of Pittsburgh (distant metastases) and Charite Universitaetsmedizin Berlin IRB (paired local recurrence) guidelines. Requirement for informed consent was waived, considering all samples were de-identified, there was no more than minimal risk to human subjects, and all tissue was obtained as part of routine clinical care.

### Cell culture and reagents

MDA-MB-134VI (MM134) (American Type Culture Collection [ATCC], Manassas, VA, USA) and SUM44/F (Asterand Bioscience, Detroit, MI, USA) cells were maintained in 1:1 DMEM (11965; Life Technologies, Carlsbad, CA, USA): L-15 (11415, Life Technologies) +10% fetal bovine serum (FBS) (26140; Life Technologies).^6^ LTED cell lines were maintained in IMEM (A10488; Life Technologies. Richter’s modification, no Phenol Red, no Gentamycin) +10% charcoal-stripped FBS. The following primers were used for qRT-PCR: FGFR4: 5′-tgcagaatctcaccttgattaca-3′, 5′-ggggtaactgtgcctattcg-3′, RPLP0: 5′-taaaccctgcgtggcaatc-3′, 5′-ttgtctgctcccacaatgaaa-3′. FGFR4 expression was normalized to RPLP0 for each of three biological replicates, before calculating fold-change relative to parental cell lines in full serum conditions. For IB, FGFR4 antibody sc-124 (Santa Cruz) was used at a 1:1000 dilution, and beta-actin (Sigma) at 1:10,000. Blots were imaged on the Olympus LI-COR system.

### FGFR4 IHC

For IHC, FGFR4 antibody MABD120 (Millipore Sigma) was used at a 1:250 dilution after antigen retrieval using heated citrate buffer, pH 6.0. Staining was detected using Envision Dual Link+ HRP Polymer and DAB (Dako). FGFR4 IHC was performed on an ER+ IDC bone metastasis collected from the University of Pittsburgh, and a paired ER+ IDC primary tumor and metachronous local recurrence collected from Charite Universitaetsmedizin Berlin. The bone metastasis was detected 41 months following primary tumor diagnosis and treatment with chemotherapy, trastuzamab, and anastrozole. The local recurrence was detected 37 months following primary tumor diagnosis and treatment with chemotherapy, trastuzamab, and tamoxifen. Additional antibody validation of MABD120 is described in the Supplementary Material, using cell lines and 22 primary ER+ ILCs.

### FGFR4 hotspot mutation rates

The FGFR4 hotspots (N535 and V550) were queried in MSK-IMPACT and the Lefebvre et al. study using the cBio portal,^29^ and MET500 using the MET500 portal (https://met500.path.med.umich.edu). MSK-IMPACT contains designations for primary and metastatic tumors, whereas Foundation Medicine contains designations for local (including local recurrences) and metastatic tumors. In all cases, lymph node metastases and distant recurrences were grouped together. For analysis of mutation rate in the Foundation Medicine and Lefebvre et al. studies, tumors of unspecified histology with a *CDH1* mutation or homozygous deletion in *CDH1* were classified as ILC. Approval for use of the Foundation Medicine data was obtained from the Western Institutional Review Board (Protocol no. 20152817).

### Statistical considerations

GraphPad Prism software version 7, and R version 3.4.1 were used for statistical analysis. All tests were two-tailed, with *p* < 0.05 considered statistically significant. Paired Wilcoxon rank signed tests were used for expression gains in metastases. Fisher’s exact tests were used to quantify odds-ratios and significance for enrichment of FGFR4 hotspot mutations.

### Reporting summary

Further information on research design is available in the Nature Research Reporting Summary linked to this article.

## Supplementary information

Supplementary Material

Supplementary Data 1

Supplementary Data 2

Reporting Summary

## Data Availability

The data generated and analyzed during this study are described in the following data record^30^: 10.6084/m9.figshare.7704371. Clinicopathologic data and FGFR4 expression for matched primary:metastatic tumors studied are available in Supplementary Data 1. Clinicopathologic data and FGFR4 hotspot mutation allele frequencies from MSK-IMPACT are available in Supplementary Data 2. Additional validation for IHC antibody and additional data comparing RNA and protein expression are available in the Supplementary Material. Raw RNA-Seq data for the paired primary and metastatic samples are not published openly in order to protect participant identities, but will be made available upon request and under regulatory compliance via a data usage agreement (DUA). For all RNA-Seq samples, the transcript counts processed via Salmon are available at https://github.com/leeoesterreich.

## Supplementary information

**Supplementary information** accompanies the paper on the *npj Breast Cancer* website (10.1038/s41523-019-0114-x).
