# Supplementary material for: FGFR4 overexpression and hotspot mutations in metastatic ER+ breast cancer are enriched in the lobular subtype
Source: NPJ Breast Cancer. 2019 Jun 27;5:19. doi: 10.1038/s41523-019-0114-x (PMC6597581; doi:10.1038/s41523-019-0114-x)
Supplement: Supplementary file 1 — Supplementary Material [file 41523_2019_114_MOESM1_ESM.pdf]

Supplementary Material

FGFR4 antibody validation

To validate the specificity of the MABD120 (Millipore Sigma) antibody for FGFR4, we first engineered Sum44PE cells that contain a doxycycline-inducible FGFR4 shRNA. Sum44PE cells were infected with an rtTA construct (a gift from Scott Lowe, PhD; Addgene#18782; modified by Dr. Lowe's laboratory), followed by infection with LT3GEPIR[1] constructs containing the following sequence within the miR-E backbone targeting FGFR4: ACGTCAAGATGCTCAAAGACAA. 100ng/ml doxycycline (dissolved in water) was added for 48 hours to induce shRNA expression. Immunoblots of FGFR4 were compared using two previously validated antibodies: sc-124 (Santa Cruz) and CST-8562 (Cell Signaling) (Supplementary Figures 1-2).

Next, immunoprecipitation of FGFR4 in MDA-MB-453 cells was performed using 2ug of rabbit IgG, 2ug of sc-124 (rabbit), or 2ug of CST-8562 (rabbit) antibodies, followed by an immunoblot of FGFR4 using the MABD120 antibody (mouse, 1:500) (Supplementary Figures 1-2).

Sum44PE (Asterand) were maintained as described previously[2] in DMEM-F12 with 2% charcoal-stripped serum and supplements. MDA-MB-453 (American Type Culture Collection [ATCC]) were cultured in DMEM (11965; Life Technologies) +10%FBS (26140; Life Technologies).

FGFR4 RNA and protein correlation

To assess the relationship between FGFR4 RNA and protein expression, we queried the RATHER consortium microarray and RPPA data of ILC primary tumors[3]. Although FGFR4 RNA was only weakly correlated with protein expression in that study, high RNA expression (upper quartile) was predictive of high protein expression (Supplementary Figure 3A-B).

We next performed IHC staining on a cohort of ER+ primary ILC tumors. From a limited sample size of 22 patients thus far, FGFR4 RNA and protein expression is significantly correlated, and again, high RNA expression (upper quartile) is predictive of high protein expression.

RATHER FGFR4 RPPA data was downloaded from GSM1626980 as log2 transformed and centered around zero. Cell Signaling antibody CST-8562 was used. FGFR4 microarray expression data was downloaded from GSE68057 as log2 normalized counts centered around zero.

For IHC of FGFR4, antibody MABD120 (Millipore Sigma) was used at a 1:250 dilution. Staining was done in a blinded manner and one representative image was captured at 200x magnification. Protein expression in tumor cells was ranked from low to high by 2 blinded observers. Ranks were then averaged between the 2 observers. FGFR4 RNA expression was assessed using the NanoString platform[4]. Sample processing was done using the automated nCounter Prep Station. Raw counts were then collected from the nCounter Digital Analyzer and transferred to the nSolver™ software (v 2.5) for data analysis. Raw counts were normalized to 5 invariant reference probes.

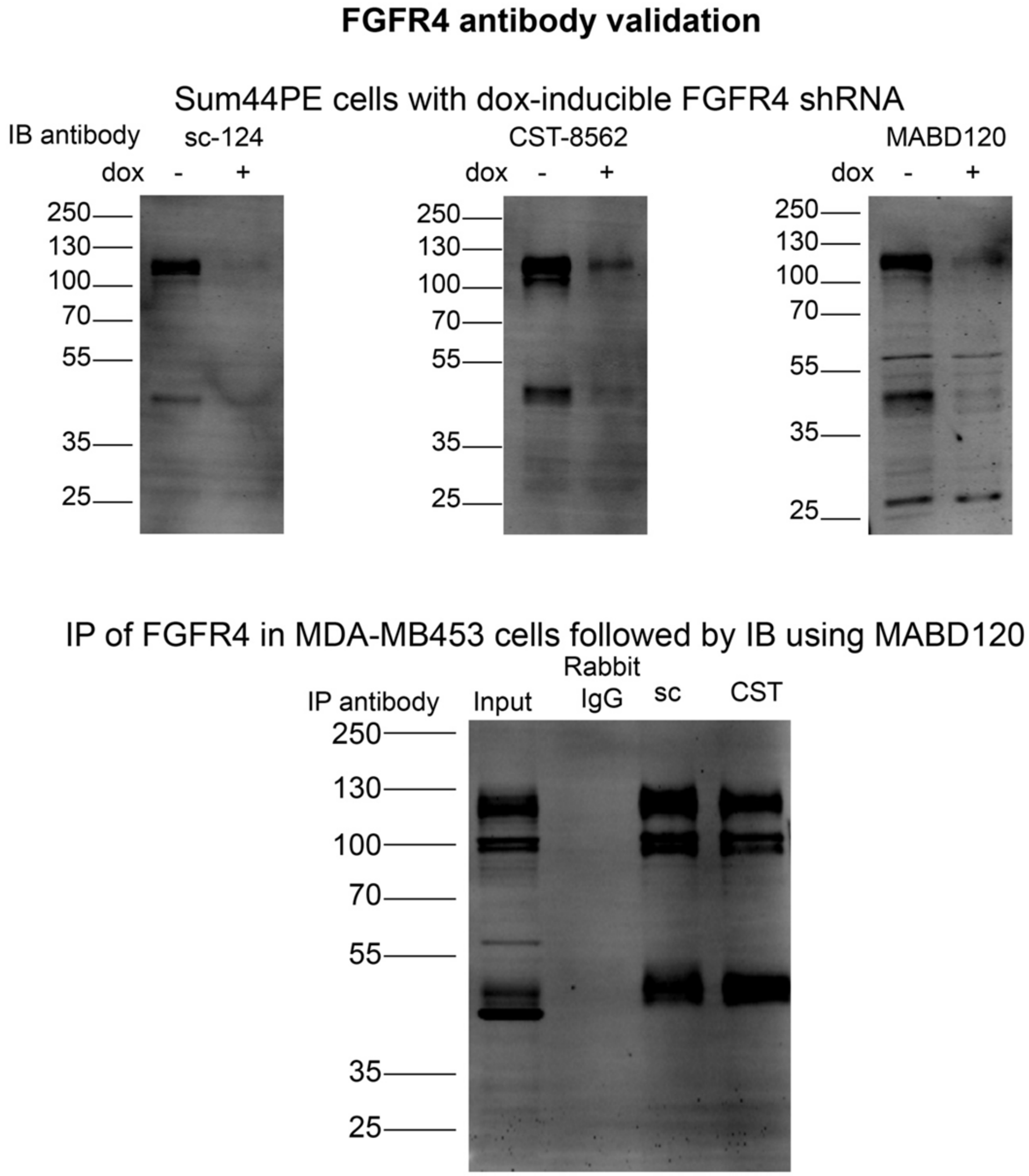

Supplementary Figure 1.
Top, FGFR4 immunoblots using sc-124, CST-8562, and MAB120 antibodies. Sum44PE cells with dox-inducible shRNA were treated with 100ng/ml dox (in water) for 48 hours. The same protein lysate was used for all 3 blots. Protein markers in kDa are shown to the left.
Bottom, immunoprecipitation (IP) using control 2ug of rabbit IgG, sc-124, or CST-8562 FGFR4 antibodies followed by immunoblot (IB) using MABD120 (1:500).

Uncropped blots of Supplementary Figure S1

Sum44PE cells with dox-inducible FGFR4 shRNA

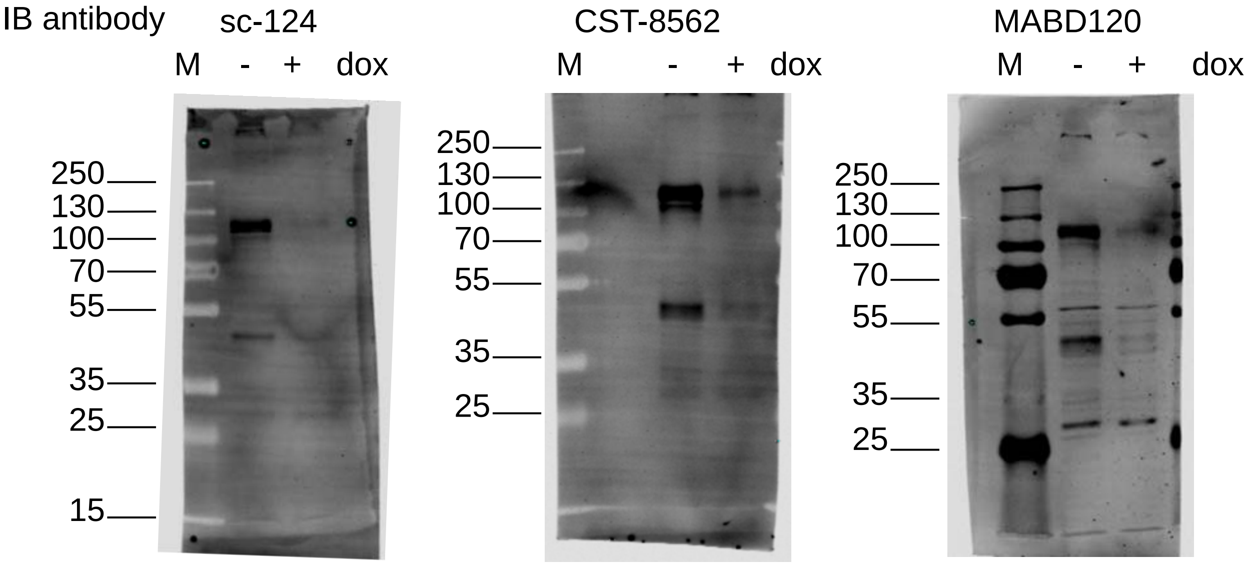

IP of FGFR4 in MDA-MB453 cells followed by IB using MABD120

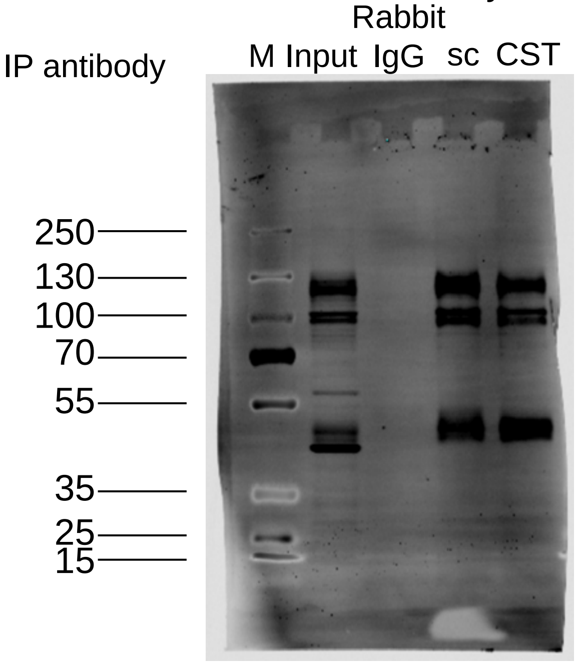

Supplementary Figure 2.
Uncropped blots of Supplementary Figure 1. M=Molecular marker, in kDa.

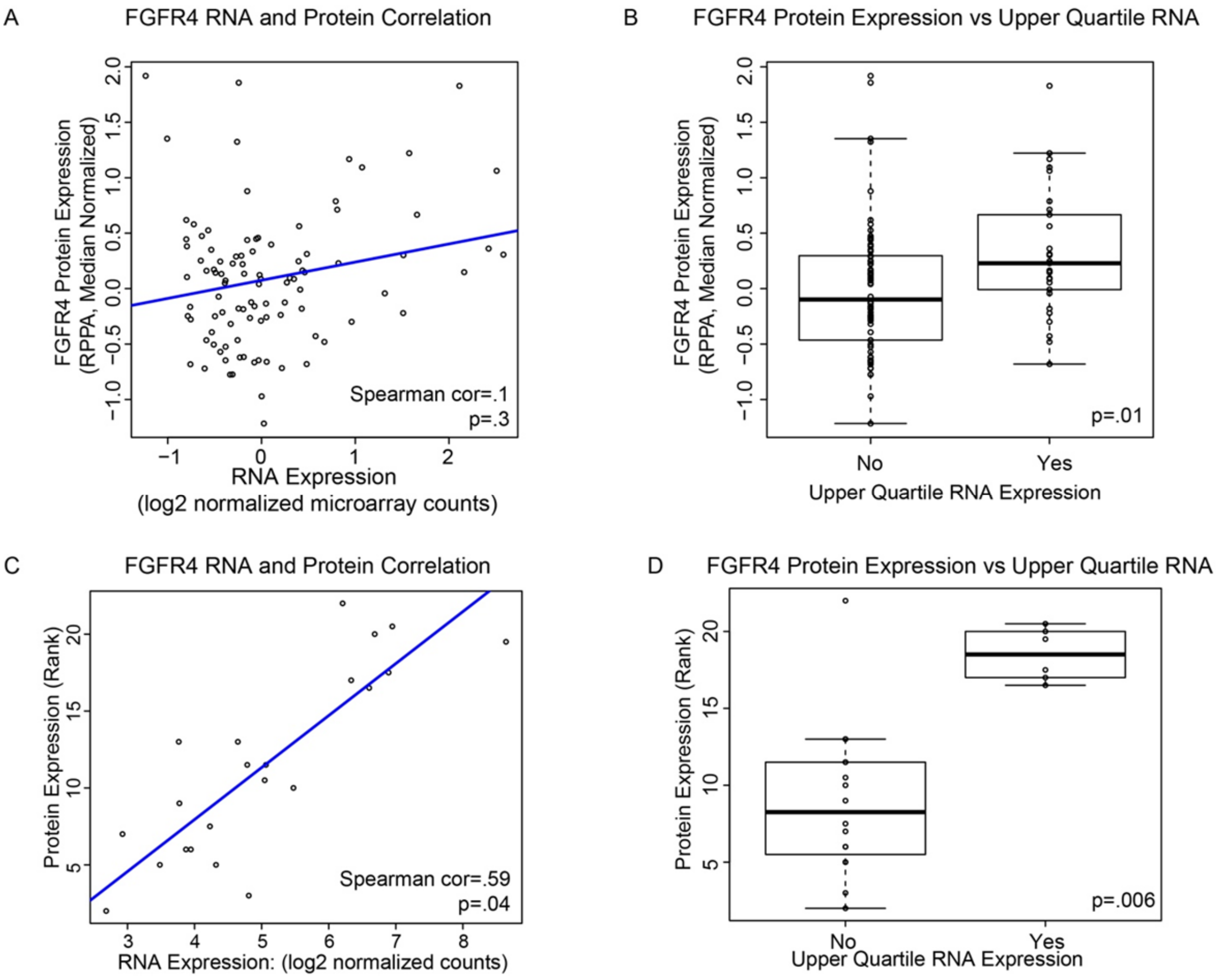

Supplementary Figure 3.
A) FGFR4 RNA and protein correlation for 99 primary ILC patients in RATHER. Spearman correlation = 0.1, p=0.3. Blue line represents linear regression fit.
B) FGFR4 protein expression in RATHER with patients separated by upper quartile FGFR4 RNA expression. Mann-Whitney U p=0.01.
C) FGFR4 RNA and protein correlation for 22 primary ILC patients from Magee-Women's Hospital. Spearman correlation = 0.59, p=0.04. Blue line represents linear regression fit. Protein expression rank from 1=lowest to 22=highest.
D) FGFR4 protein expression from Magee-Women's Hospital patients separated by upper quartile FGFR4 RNA expression. Mann-Whitney U p=0.006.

References

1. Fellmann, C. *et al.* An optimized microRNA backbone for effective single-copy RNAi. *Cell Rep.* **5,** 1704–1713 (2013).
2. Sikora, M. J. *et al.* Invasive lobular carcinoma cell lines are characterized by unique estrogen-mediated gene expression patterns and altered tamoxifen response. *Cancer Res.* **74,** 1463–1474 (2014).
3. Michaut, M. *et al.* Integration of genomic, transcriptomic and proteomic data identifies two biologically distinct subtypes of invasive lobular breast cancer. *Sci. Rep.* **6,** 18517 (2016).
4. Geiss, G. K. *et al.* Direct multiplexed measurement of gene expression with color-coded probe pairs. *Nat. Biotechnol.* **26,** 317–325 (2008).
